# Veterinary Department, McGill University

**Published:** 1895-08

**Authors:** 


### Veterinary Department, McGill University.

The Veterinary Department of McGill University will open her 1895–'96 term on September 24th, and continue until the end of March, making the full six months' course.

The matriculation examination, always high at McGill, will be shortly advanced to include the rudiments of Latin.

The Council of Agriculture, in consideration of aid annually granted the school, has the privilege of enrolling thirteen pupils, the same to be given to deserving students, and only those who fully intend completing the course.

